# Multicentre analysis of intensity of care at the end-of-life in patients with advanced cancer, combining health administrative data with hospital records: variations in practice call for routine quality evaluation

**DOI:** 10.1186/s12904-019-0419-4

**Published:** 2019-04-05

**Authors:** Isabelle Colombet, Carole Bouleuc, Alain Piolot, Aurélie Vilfaillot, Hélène Jaulmes, Sabine Voisin-Saltiel, François Goldwasser, Pascale Vinant, the EFIQUAVIE study group, Jérôme Alexandre, Muriel Mons, François Hemery, Samir Bouam, Ilhem Cherrak, Gilles Chatellier

**Affiliations:** 1 Unité Fonctionnelle de Médecine Palliative, Hôpital Cochin, Assistance Publique Hôpitaux de Paris, F-75014 Paris, France; 2 Univ Paris Descartes, F-75006 Paris, France; 3 Département de Soins de Support, Institut Curie, Paris, France; 4 Unité Mobile d’Accompagnement et de Soins Palliatifs, Hôpital Henri Mondor, Assistance Publique Hôpitaux de Paris, F-94000 Créteil, France; 5 Unité de Recherche Clinique, Hôpital européen G Pompidou, Hôpitaux Universitaire Paris Ouest, Assistance Publique Hôpitaux de Paris, F-75015 Paris, France; 6 Unité Mobile d’Accompagnement et de Soins Palliatifs, Hôpital européen G Pompidou, Hôpitaux Universitaire Paris Ouest, Assistance Publique Hôpitaux de Paris, F-75015 Paris, France; 7 Unité Mobile d’Accompagnement et de Soins Palliatifs, Institut Gustave Roussy, Villejuif, France; 8 Oncologie, Hôpital Cochin, Hôpitaux Universitaire Paris Centre, Assistance Publique Hôpitaux de Paris, F-75014 Paris, France

**Keywords:** (MesH heading or entry terms), End of life care, Quality of health care, Palliative care, Cancer care facilities, Academic medical centers, Data collection methods

## Abstract

**Background:**

Accessible indicators of aggressiveness of care at the end-of-life are useful to monitor implementation of early integrated palliative care practice. To determine the intensity of end-of-life care from exhaustive data combining administrative databases and hospital clinical records, to evaluate its variability across hospital facilities and associations with timely introduction of palliative care (PC).

**Methods:**

For this study designed as a decedent series nested in multicentre cohort of advanced cancer patients, we selected 997 decedents from a cohort of patients hospitalised in 2009–2010, with a diagnosis of metastatic cancer in 3 academic medical centres and 2 comprehensive cancer centres in the Paris area. Hospital data was combined with nationwide mortality databases. Complete data were collected and checked from clinical records, including first referral to PC, chemotherapy within 14 days of death, ≥1 intensive care unit (ICU) admission, ≥2 emergency department visits (ED), and ≥ 2 hospitalizations, all within 30 days of death.

**Results:**

Overall (min-max) indicator values as reported by facility providing care rather than the place of death, were: 16% (8–25%) patients received chemotherapy within 14 days of death, 16% (6–32%) had ≥2 admissions to acute care, 6% (0–15%) had ≥2 emergency visits and 18% (4–35%) had ≥1 intensive care unit admission(s). Only 53% of these patients met the PC team, and the median (min-max) time between the first intervention of the PC team and death was 41 (17–112) days. The introduction of PC > 30 days before death was independently associated with lower intensity of care.

**Conclusions:**

Aggressiveness of end-of-life cancer care is highly variable across centres. This validates the use of indicators to monitor integrated PC in oncology. Disseminating a quality audit-feedback cycle should contribute to a shared view of appropriate end-of-life care objectives, and foster action for improvement among care providers.

## Background

Despite increased survival following advances in early detection and treatment, numbers of deaths from cancer are expected to increase as a result of the ageing population. Efforts to improve the quality of end-of-life cancer care are therefore important. One of the challenges for quality management is the efficient measurement of care quality with rapid feedback to healthcare organizations enabling them to undertake necessary actions for improvement. Earle et al. [1] developed indicators involving focus groups with patients, carers and health professionals, designed to be easily accessible and measurable from health administrative data and to provide meaningful information on the quality of end-of-life cancer care. These indicators describe high-intensity medical care delivered in the last month of life, such as overuse of chemotherapy, underuse of hospice care, frequent hospitalizations, emergency room visits, and intensive care unit admissions. They set some achievable benchmarks from the results of the 10% best-performing providers [2–4]. The methodology was also tested in Canada [5], and the indicators were endorsed by the American National Quality Forum [3]. Since these original developments, and with the computerization of clinical activities and easier access to large health administrative databases, these indicators have been used in other countries [6–8], in child populations [8, 9], or for specific types of cancer [10, 11]. In France, a study described the chemotherapy indicator, using nation-wide hospital administrative data, primarily collected for hospital payment without recording outpatient care [12]. In another study, all Earle’s indicators were used to evaluate the effect of integrated palliative care on the quality of end-of-life care from exhaustive data in an academic medical centre [13].

Based on evidence from several randomized clinical trials, American Society of Clinical Oncology (ASCO) as well as European Society of Medical Society (ESMO) published guidelines to recommend patients and outpatients with advanced cancer should receive dedicated palliative care services, early in the course of disease, concurrent with active treatment [14, 15]. A Cochrane meta-analysis confirmed that early palliative care could improve quality of life and reduce symptom intensity with no effect reaching statistical significance on survival [16]. Some studies found that early palliative care also had a favorable impact on end-of-life care aggressiveness, suggesting that such indicators as chemotherapy administration or intensive care resource use can be considered as interesting to monitor implementation of early palliative care practice [17–21].

In this multicentre study, we selected a cohort of patients diagnosed with metastatic cancer in 5 academic medical centres or comprehensive cancer centres. The study aimed to determine the intensity and trajectory of end-of-life cancer care from exhaustive data combining administrative databases and hospital clinical records, to evaluate their variability across hospital facilities and their association with timely integration of palliative care.

## Methods

### Design and setting

We conducted a retrospective analysis of a nested series of decedents in a cohort selected from the administrative data of 2 comprehensive cancer centres and 3 academic medical centres in the Paris region. All 2010 decedents were identified from these hospitals’ administrative databases and by linkage with national death certificates database, to analyse quality indicators for all inpatients diagnosed with advanced cancer, whatever their place of death. Quality indicators were then measured from hospital administrative database, completed by data collected from health records. The REporting of studies Conducted using Observational Routinely collected health Data (RECORD) guidelines were followed when relevant to report methods and results [22].

Organisation of the hospital-based palliative care consultation team is similar in each centre. The team comprises at least a palliative care physician and a palliative care nurse who collaborate systematically with social workers and psychologists. They can be called on by attending physicians to evaluate in- or outpatients, give advice on symptom relief, and provide support for carers or healthcare professionals.

None of the participating centres has an inpatient palliative care unit.

### Data sources and study population

Patients over 18 years of age were selected from each hospital administrative database (*PMSI* database, the French equivalent of DRG database), based on a hospital stay coded under metastatic cancer ICD-10 diagnosis (C76_, C78_, C79_, C80_), between October 1, 2009 and December 31, 2010. Patients recorded as deceased in 2010 the database of one of the facilities were identified. For patients whose death was not found in the hospital administrative database on the date of the request, a vital status search was performed on the National Vital Statistics (*RNIPP, for National Register for the Identification of Private Individuals*) database, by application to the national death certificate database to obtain the cause and place of death [23, 24]. All 2010 decedents in the initial cohort selected from hospital administrative databases were thus identified, whatever their place of death. Two hundred patients per centre were randomly selected from this decedent series, stratified according to age, gender and place of death (in hospital or elsewhere), to form a sample of 1000 patients, equally spread across the 5 participating centres and representative of each.

### Measures of intensity of care

For the 200 patients selected in each centre, a second request to each hospital administrative database was made to enable the reconstruction of clinical trajectories in the last month of life: the number of visits to emergency room or oncology clinic, the number of admissions to intensive care unit or to acute care.

### Additional data collected from clinical records

The data extracted from hospital administrative database was systematically checked and completed individually for each patient by a search of hospital clinical records in each participating centre. Additional data described the exact date of the first intervention of the palliative care team, and, when available, the modalities of last administered chemotherapy (route and exact date of prescribing). We also checked clinical records to appreciate whether the centre where the patient was identified was the patient’s reference centre for cancer treatment, and the length of the patient’s follow-up in that centre. The study protocol was approved by the CEERB (N° IRB00006477).

### Statistical analyses

Quantitative and qualitative variables were described by means (SD) and frequencies (%). For results per centre, each patient was reported in the centre where he/she was identified by request to the hospital administrative database. First, Chi-square and Student tests were performed to assess the associations between outcomes (i.e. measures of intensity of care) and the following covariates: age at death, gender, disease incurable at initial diagnosis, number of metastasis sites, study centre, intervention of palliative care team, group of primary tumor sites (defined in 3 categories of expected survival according to published French epidemiological data) at least one admission in an intensive care unit, at least one admission in acute care, at least one emergency visit. Then, logistic regressions were used to predict the logit of the probability of experiencing each outcome. Since the outcome indicators of quality of end of life care include the timeframe of the last 30 days of life (e.g. emergency visit, intensive care unit or acute care admission in the last 30 days of life), we represented the timely intervention of palliative care team variable as “Early intervention of palliative care team (> 30 days before death)” versus “No or late intervention of palliative care team (< 30 days before death)”. Variables with a *p*-value under 0.05 in the simple analysis were included in the multivariable analysis, making analysis of the effect of palliative care within the same timeframe of the last 30 days of life difficult. In addition, we conducted some sensitivity analysis only for the place of death outcome, in order to test the question of palliative care versus no palliative care (using other representation of “No intervention” versus “Intervention of palliative care”), separately from the question of early versus late palliative care.

All statistical tests were two sided and a *p*-value under 0.05 was considered statistically significant. All analyses were performed using SAS software version 9.4.

## Results

### Study population and patient characteristics per Centre

**Fig. 1:**
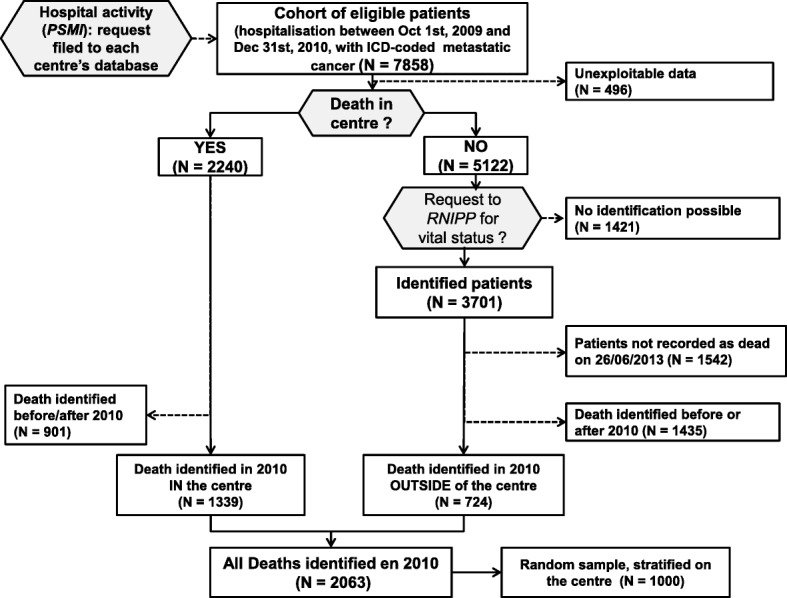
Flow chart of study

A total of 7858 patients were hospitalized with a diagnosis of metastatic cancer between October 2009 and December 2010 in the 5 participating centres, among whom 2063 were identified as decedents in 2010 (see Fig. 1); 724 (35%) patients who died outside hospital were identified thanks to the request to *RNIPP*. From these 2063 patients, a random sample of 1000 patients was drawn to pursue data collection from clinical records. Three patients were excluded from the analysis due to identity or primary diagnosis errors.

Men accounted for 54%, mean age 66 (±14.2) years at the time of death (Table 1). The most frequent primary tumor sites were breast (18%), lung (17%), urogenital (prostate, bladder, kidney, 14%) and colon/rectum (11%). In the study population, 599 (66%) patients had ≥2 metastatic sites, most frequently liver (48%), bone (43%), lung (42%), peritoneal (20%) and brain (16%).

**Table 1:** Patient characteristics by centre

	TOTAL *n* = 997	UH 1 *n* = 200	CCC 1 *n* = 200	UH 2 *n* = 199	CCC 2 *n* = 200	UH 3 *n* = 198
Age at death, *mean (SD)*	*66* (14)	*68* (13)	*64* (14)	*70* (13)	*60* (14)	*70* (13)
Men, n (%)	535 (54)	130 (65)	42 (21)	126 (63)	111 (56)	126 (64)
Primary tumor site, n (%)	*n* = 976	*n* = 198	*n* = 200	*n* = 197	*n* = 200	*n* = 181
Breast	173 (18)	12 (6.1)	106 (53)	15 (7.6)	24 (12)	16 (8.8)
Lung	165 (17)	32 (16)	27 (14)	48 (24)	40 (20)	18 (9.9)
Urinary tract and kidney	136 (14)	31 (16)	9 (4.5)	40 (20)	19 (9.5)	37 (20)
Colorectal	104 (11)	21 (11)	9 (4.5)	24 (12)	23 (12)	27 (15)
Liver, Pancreas, Biliary tract	89 (9.1)	31 (16)	4 (2.0)	15 (7.6)	10 (5.0)	29 (16)
Other	309 (32)	71 (36)	45 (23)	55 (28)	84 (42)	54 (30)

*Abbreviations: CCC* Comprehensive Cancer Centre, *UH* University Hospital

### Intensity of end-of-life care

All indicators were highly variable across centres (Table 2).

**Table 2:** Intensity in end-of-life care, per centre

	TOTAL		UH 1		CCC 1		UH 2		CCC 2		UH 3	
	n	(%)	n	(%)	n	(%)	n	(%)	n	(%)	n	(%)
Chemotherapy in last 14 days of life	116/738	(15.7)	15/126	(11.9)	30/185	(16.2)	21/159	(13.2)	42/169	(24.9)	8/99	(8.1)
Trajectory of care in last month of life	*n* = 997		*n* = 200		*n* = 200		*n* = 199		*n* = 199		*n*= 198	
1 admission in acute care	520	(52.2)	101	(50.5)	92	(46.0)	113	(56.8)	103	(51.5)	111	(56.1)
≥ 2 admissions in acute care	164	(16.4)	27	(13.5)	35	(17.5)	27	(13.6)	64	(32.0)	11	(5.6)
1 emergency visit	197	(19.8)	57	(28.5)	11	(5.5)	60	(30.2)	89	(44.5)	41	(20.7)
≥ 2 emergency visits	61	(6.1)	9	(4.5)	0	**.**	13	(6.5)	29	(14.5)	10	(5.1)
≥ 1 admission in Intensive Care Unit	174	(17.5)	35	(17.5)	8	(4.0)	38	(19.1)	23	(11.5)	70	(35.4)
Patients transferred in palliative care unit	*n* = 157		*n* = 33		*n* = 58		*n* = 29		*n* = 10		*n* = 19	
≤ 3 days before death	12	(7.6)	0	**–**	3	(5.2)	6	(20.7)	0	**–**	3	(15.8)
Place of Death	*n* = 978		*n* = 199		*n* = 189		*n* = 199		*n* = 198		*n* = 193	
Acute care hospital	672	(68.7)	129	(64.8)	95	(50.3)	139	(69.8)	182	(91.9)	127	(65.8)
Acute care ward	583	(59.6)	113	(56.8)	93	(49.2)	115	(57.8)	146	(73.7)	116	(60.1)
Intensive Care Unit	62	(6.3)	15	(7.5)	2	(1.1)	22	(11.1)	17	(8.6)	6	(3.1)
Emergency room	27	(2.8)	1	(0.5)	0	.	2	(1.0)	19	(9.6)	5	(2.6)
Palliative care unit	189	(19.3)	41	(20.6)	60	(31.7)	32	(16.1)	10	(5.1)	46	(23.8)
Home	82	(8.4)	24	(12.1)	13	(6.9)	24	(12.1)	4	(2.0)	17	(8.8)
Other	35	(3.6)	5	(2.5)	21	(11.1)	4	(2.0)	2	(1.0)	3	(1.6)

*Abbreviations*: *CCC* Comprehensive Cancer Centre, *UH* University Hospital

Of the 738 patients for whom the data could be found in hospital records, 16% received chemotherapy in their last 14 days of life (Table 2). This proportion varied across centres, from 8.1 to 13.2% in the three academic medical centres and reached 16 and 25% in Comprehensive Cancer Centres 1 and 2. The last line of chemotherapy was started at a median of 42 days preceding death. It was prescribed by oral route for 54/236 (23%) patients.

Concerning clinical trajectories in the last month before death, in the overall study population, 16.4% were hospitalized twice or more (reaching 32% for Comprehensive Cancer Centre 2) and 90% of these admissions were motivated by needs for palliative or supportive care.

Sixty-one (6%) patients visited emergency room twice or more. Only 10 (3.4%) of these visits led to hospitalisation. In all 17.5% were admitted at least once to intensive care unit, with a median length of stay of 4 days. Between-centre variations for these indicators should be interpreted bearing in mind that Comprehensive Cancer Centre 1 has no emergency room, and is organized to receive patients in need of urgent care in unplanned consultations, or addresses them to the emergency room in a nearby public hospital.

### Impact of palliative care on intensity of end-of-life care

The median anteriority of follow up differed across centres and the timing of referral to palliative care should be read in this context (Table 3). Both comprehensive cancer centres were considered as the referent centre for more than 90% of the patients (respectively 93 and 98%) and a large majority had been followed for over 6 months. In the overall population, the palliative care team was mobilized for 492 (53%) patients, the proportion ranging from 30% in academic medical centre 2 to 70% in comprehensive cancer centre 1.

**Table 3:** Clinical trajectory and context of referral to palliative care

	TOTAL	UH 1	CCC 1	UH 2	CCC 2	UH 3
Study centre is referent for the patient’s cancer, n/total (%)	842/932 (90)	181/200 (91)	196/200 (98)	163/199 (82)	187/200 (94)	115/133 (87)
Anteriority of follow up in the centre, *Median time in months (Q1 - Q3)*	*12 (4–37)*	*10* (4–24)	*33 (9–106)*	*8* (3–21)	*17 (6–43)*	*8* (2–21)
Intervention by the Palliative Care Team, n/total (%)	492/926 (53)	101/196 (52)	140/199 (70)	59/196 (30)	112/199 (56)	81/136 (60)
ECOG PS at 1st intervention ≤2	95/349 (27)	23/67 (34)	43/83 (52)	5/21 (24)	12/101 (12)	12/77 (16)
Time between first intervention and date of death	*n* **=** 475	*n* = 98	*n* = 138	*n* = 55	*n* = 107	*n* = 77
≤ 7 days	81 (17)	12 (12)	10 (2, 7)	10 (18)	32 (30)	17 (22)
]7–30] days	117 (25)	15 (15)	19 (14)	22 (40)	35 (33)	26 (34)
]30–90] days	128 (27)	39 (40)	31 (23)	14 (26)	24 (22)	20 (26)
> 90 days	149 (31)	32 (33)	78 (57)	9 (16)	16 (15)	14 (18)
median (Q1 - Q3)	41 (13–122)	63 (25–115)	112 (38–281)	25 (11–50)	17 (7–54)	21 (8–64)

*Abbreviations*: *CCC* Comprehensive Cancer Centre, *UH* University Hospital

Table 4 shows the results of the multivariable logistic regression analyses. The intervention of a palliative care team more than 30 days before death was associated with lower likelihood of receiving chemotherapy near death (OR 0.50 [IC95% 0.30–0.82]), of being admitted to acute care in the last month (OR 0.64 [IC95% 0.46–0.89]), and of dying in acute care unit (OR 0.33 [IC95% 0.23–0.47]). According to sensitivity analyses for the place of death outcome, the intervention of palliative care team, whenever its timing, as compared with no intervention, was still significantly associated with more frequent dying in acute care unit (OR 0.40 [IC95% 0.28–0.57], *p* < 0.0001) .

**Table 4:** Unadjusted frequencies of each indicator by delivery of palliative care and multivariable logistic regression predicting intensity of care near death

	Early intervention of PCT (> 30 days before death)		No or late intervention of PCT (< 30 days before death)		Multivariable analysis^a^		
Indicators	n	(%)	n	(%)	OR	IC95%	*p*-value
Chemotherapy in last 14 days of life	28/240	(11.7)	89/487	(18.3)	0.50	[0.30–0.82]	0.006
≥ 1 emergency visits	62/282	(22.0)	188/644	(29.2)	1.04	[0.72–1.49]	0.844
≥ 1 admission in ICU	49/282	(17.4)	114/644	(17.7)	1.45	[0.95–2.21]	0.082
≥ 1 admission in acute care	180/282	(63.8)	481/644	(74.7)	0.64	[0.46–0.89]	0.009
Place of death in acute care hospital	153/273	(56.0)	511/637	(80.2)	0.33^b^	[0.23-0.47]	< 0.0001

*Abbreviations*: *PCT* Palliative Care Team, *ICU* Intensive Care Unit

^a^Odds Ratio of indicator, according to the timing of intervention of PCT, adjusted on age at death, gender, disease incurable at initial diagnosis, number of metastasis sites, group of primary tumor sites (defined in 3 categories of expected survival according to published French epidemiological data), and study centre

^b^also adjusted on previous indicators of intensity of care

No other covariable was significantly associated with intensity of care, except the centre and older age. The centre was significantly associated with all indicators, and older age only associated with less chemotherapy near death (OR 0.97 [IC95% 0.96–0.98]).

## Discussion

This study provides exhaustive baseline data on the intensity and trajectory of end-of-life care delivered to adult populations with metastatic cancer in two comprehensive cancer centres and three academic hospitals in the Paris area. Its results highlight some inter-centre variability of practice, with max/min ratios between 3 and 9, depending on indicators. Hardly more than half patients met the hospital-based palliative care consultation team. Adjusting for centre effect, the intervention of the palliative care team more than a month before death was significantly associated with lesser likelihood of patients receiving chemotherapy near death and greater likelihood of dying in palliative care unit or at home.

Results obtained from large nationwide health administrative data are interesting to give a broad view of practice. Studies of these indicators from other countries reveal large variations which can be explained by differences in healthcare systems and public health policies at the national level, and by heterogeneity in measurement methods and data sources [4, 6, 12, 25–27]. Our findings remain in the broad range of results published. It is necessary for a good performance measure to detect differences in quality of care. We obtained a significant variability of practice between the 5 participating centres, similar to the results of Earle et al. whose ratios between the 5% best and 5% worst performing health care geographic areas ranged from 2.2 to 5, according to outcome [2].

We found high frequencies of chemotherapy administration in the 14 days before death, with overuse more frequent in comprehensive cancer centres than in academic medical centres and higher intensity of care among young patients, as previously reported in the French national hospital administrative database [12].

Another strength of this study is the collection of data from clinical records giving access to the accurate timing of the first intervention of the palliative care team which is not recorded in *hospital administrative* database [28]. This allowed to describe large variations in the timing of referral to palliative care teams across centres and to analyse the accurate association between this intervention and the indicators of intensity of care. These variations suggest that early palliative care, known to improve quality-of-life [16] and internationally recommended for patients with advanced cancer [14, 15], is unequally put into practice. More systematic monitoring of the median time between first referral to palliative care and death as an indicator of this practice could provide interesting leverage for change [29, 30].

However, the study cohort was recruited from academic medical centres and comprehensive cancer centres only, making overall results on indicators not representative of all patients with advanced cancer. As one centre (comprehensive cancer centre 1) is specialized in breast cancer treatment, and another (academic medical centre 1) is an expert centre for the diagnosis and treatment of sarcoma, women and young patients were over-represented. We found a particularly high rate of patients admitted at least once in intensive care unit during their last month (18%) and the proportion of deaths at home observed in our study is lower than that reported nationally for cancer deaths (8% versus 19%) [31]. In their European study from death certificates, Cohen et al. give some insights into between-country variations concerning place of death and quality indicator results. French healthcare organization is characterized by a high proportion of people dying in hospital, especially from cancer (> 70%), alongside one of the highest ratios per 10,000 of both acute care hospital beds and long term care beds. Among the other 8 European countries participating in the study, France also has the highest healthcare and social welfare expenditure, with the lowest rates of palliative care services for adults per million inhabitants.

## Conclusion

This study brings a first multicentre measure of all Earle’s indicators in French setting, from health administrative data completed by hospital clinical records. It supports a prospective approach to quality of care, reporting indicators of practice from the point of view of cancer care providers, by facility providing care rather than the place of death. Unlike results obtained from large health administrative databases which bring a macroscopic view of practice at the national level, our approach provide practitioners with the opportunity to reflect on their practice knowing their own specificities and organization, and to engage in a quality evaluation-improvement cycle at each centre level [32]. Our results also add to those other practice evaluation studies which found that early palliative care could have a favourable impact on end-of-life care aggressiveness [20, 21]. This suggests that routine measure of such indicators as chemotherapy administration, acute care resource use in the last month of life and timing of first referral to specialized palliative care should be recommended to monitor actual implementation of early palliative care practice and end-of-life care quality at health care facility level.
